# Stigma: Lessons from Women^1^

**DOI:** 10.3201/eid1011.040624_07

**Published:** 2004-11

**Authors:** Wanda K. Jones, Diana Weil, Jeannine Coreil, Bronwyn Shoush

**Affiliations:** * Department of Health and Human Services, Washington, D.C., USA;; † World Bank, Washington, D.C., USA;; ‡ World Health Organization, Geneva, Switzerland;; § University of South Florida, Tampa, Florida, USA;; ¶ Aboriginal Justice Initiative, Edmonton, Alberta, Canada

**Keywords:** Stigma, women, Tuberculosis, Lymphatic Filariasis, Aboriginal Communities

Societies worldwide tend to stigmatize diseases associated with poverty, deviant or morally sanctioned behavior, contagion, frightening or unusual symptoms, and physical disfigurement or impairment. Stigma can lead to discrimination, shame, and fear. Stigma may be especially acute for women: it may limit their marital prospects; constrain their participation in community, household and family roles; and diminish their quality of life. For already-stigmatized communities, the added impact of infectious diseases can hinder economic and human development.

## Tuberculosis (TB): Can New Strategies To Expand Care Reduce Stigma?

Globally, TB killed an estimated 700,000 women in 2001. Men comprise 70% more of reported TB case-patients than do women, and the prevalence of disease is greater among men, except in women's early reproductive years. Gender-specific research is still limited, but studies suggest that men are less likely to complete treatment than are women. Women are more likely to seek later treatment with advanced disease. Malnutrition, HIV, civil and economic crises, and urbanization likely increase women's risk for exposure and disease. The severity of stigma varies but does affect involvement of providers, patients, and communities. Evidence from South Asia, Africa, and Vietnam suggests that the potential for stigmatization affects women's help-seeking more than men and is linked to fears of contagion and social isolation. A new global scale effort to control TB has begun, based on the directly observed treatment short course (DOTS), with over 180 countries now engaged. Still, TB case detection is improving too slowly. Measures to improve access may help alleviate stigma and enable women's engagement National TB programs and partners are pursuing community-based care, public-private provider collaboration, enablers and social support, and new social mobilization and communication strategies. Few models have gender-specific components, but these may be needed in areas where stigma is great.

## Lymphatic Filariasis

Leprosy, onchocerciasis, lymphatic filariasis, leishmaniasis, and polio are well known as disfiguring infectious diseases. In the case of lymphatic filariasis, filarial blockage of lymphatic ducts causes lymphedema, or elephantiasis in its most extreme form. The lymphatic system anywhere in the body can be involved, although women have 5–10 times the risk of their legs being affected. Odor and sores from bacterial infections, secondary to the poor circulation and skin folding further, complicate the disfiguring effects. Before a national lymphatic filariasis program was undertaken in Haiti, women there requested lymphatic filariasis support groups be established to help educate women about the disease, enable self-care regimens, enhance self-esteem and coping, promote community awareness and acceptance of the condition, and serve as a base for community development. Support groups met regularly and were connected to clinical services being provided to the women. Women participating in the groups reported fewer acute attacks, less difficulty living with lymphatic filariasis (standing, wearing shoes, inability to work, depression, shame, embarrassment), improved understanding of the vector cause, and better adherence to home care practices. Integrating social support groups with clinical treatment or eradication programs can improve outcomes for women.

## Aboriginal Communities

Homelessness, poverty, and isolation of aboriginal populations on reservations are the legacy of settlement and colonization of the New World. Newcomers brought smallpox, measles, polio, and TB, all of which decimated aboriginal populations throughout the Western Hemisphere. Even in more modern times, TB persists among some aboriginal populations, and sexually transmitted diseases and HIV now add to the diseases associated with low income and other health risks such as tobacco use and obesity. Aboriginal populations, who have witnessed their own decimation from infectious diseases brought by settlers, may not trust that what the government provides is necessarily good for them. Canada has undertaken a restorative justice initiative to enable communities to build confidence in their own capacity to improve their health. Canadian aboriginal communities applying these principles are working to restore harmony outside the justice system. They are examining national policies for potential adverse impact. Privacy policies are a good example; in a community with a group rights perspective, that community's right to be safe, secure, and healthy overrides a person's right to privacy.

